# Supplementary material for: Circuit asymmetries underlie functional lateralization in the mouse auditory cortex
Source: Nat Commun. 2019 Jun 25;10:2783. doi: 10.1038/s41467-019-10690-3 (PMC6592910; doi:10.1038/s41467-019-10690-3)
Supplement: Supplementary file 1 — Supplementary Information [file 41467_2019_10690_MOESM1_ESM.pdf]

Supplemental Information

Circuit Asymmetries Underlie Functional Lateralization in the Mouse Auditory Cortex

Levy et al.

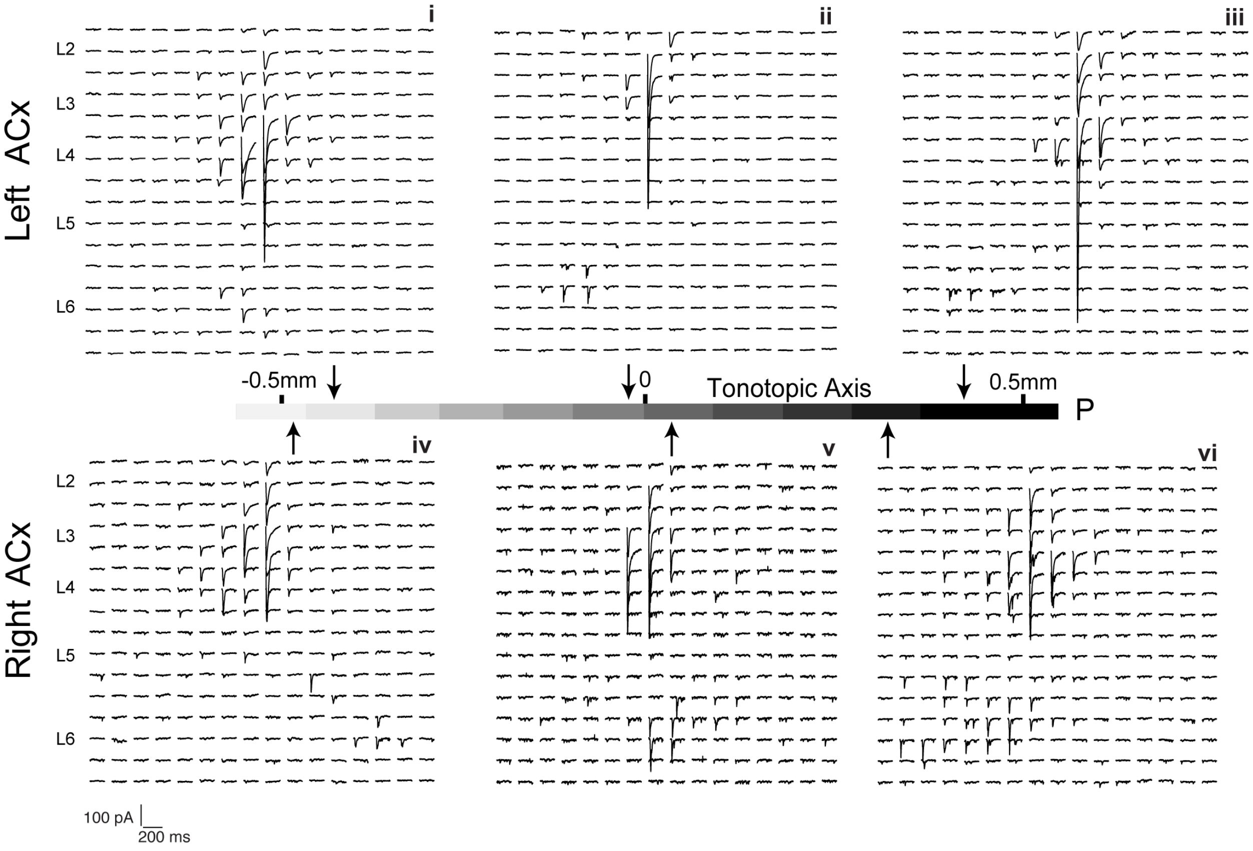

Supplementary Figure 1

Raw input traces corresponding to individual cell examples in Fig. 3B of the main text.

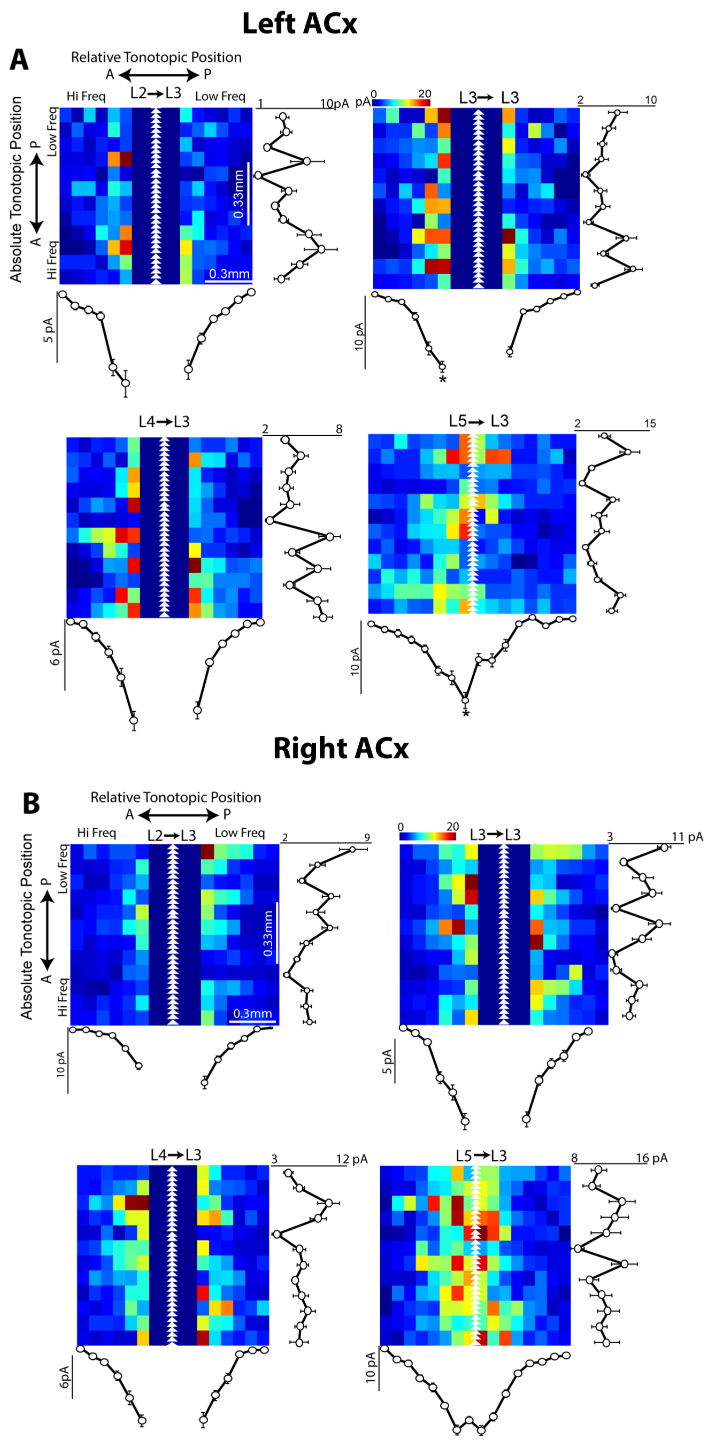

Supplementary Figure 2

Population 2D maps for all laminar pathways presynaptic to L3 (except for L6, which is found in the main figures). (**A**) Tonotopically organized laminar input presynaptic to L3 in the left ACx. Asterisks mark significant anterior-posterior biases, which were found in the input from L5 ($p = 0.03$, $n = 38$, Wilcoxon rank-sum) and L3 ($p = 0.04$, $n = 38$, Wilcoxon rank-sum). (**B**) Tonotopically organized laminar input presynaptic to L3 in the right ACx. All data are presented as mean±SEM.

Table 1

	Left ACx			Right ACx		
Pathway	slope	corr	Ant-Post p-val	slope	corr	Ant-Post p-val
L2 → 3	-0.37	-0.21	0.71	0.46	0.25	0.37
L3 → 3	-0.45	-0.17	***0.04***	-0.27	-0.22	0.8
L4 → 3	-0.48	-0.28	0.3	-0.03	-0.006	0.34
L5 → 3	0.12	0.06	***0.03***	-0.36	-0.24	0.54
L6 → 3	-1.5	***-0.72***	***1.4x10-3***	-1	***-0.71***	0.37

Table 2

Intrahemispheric Comparison: **Left ACx**
one-way ANOVA, F(2, 36) = 18.4, p < 7e-6, n = 38

95% confidence interval	**Significant**		Non-significant
	Anterior-Middle	Anterior-Posterior	Middle-Posterior
Upper bound	187.87	219.40	93.30
Lower bound	61.76	83.61	-39.92

Table 3

Intrahemispheric Comparison: **Right ACx**
one-way ANOVA, F(2, 38) = 29.82, p < 6e-7, n=40

95% confidence interval	**Significant**		
	Anterior-Middle	Anterior-Posterior	Middle-Posterior
Upper bound	326.22	532.96	355.7
Lower bound	46.38	271.2	75.86

Supplementary Tables 1-3

Summary of tonotopic statistics. (**1**) Statistical analysis of 2D average population data. Numbers in bold are statistically significant. (**2, 3**) Intrahemispheric statistical analysis of the "hotspot distance from soma" measurements. Cells were divided in three clusters spanning the tonotopic axis (Anterior, Middle, Posterior).

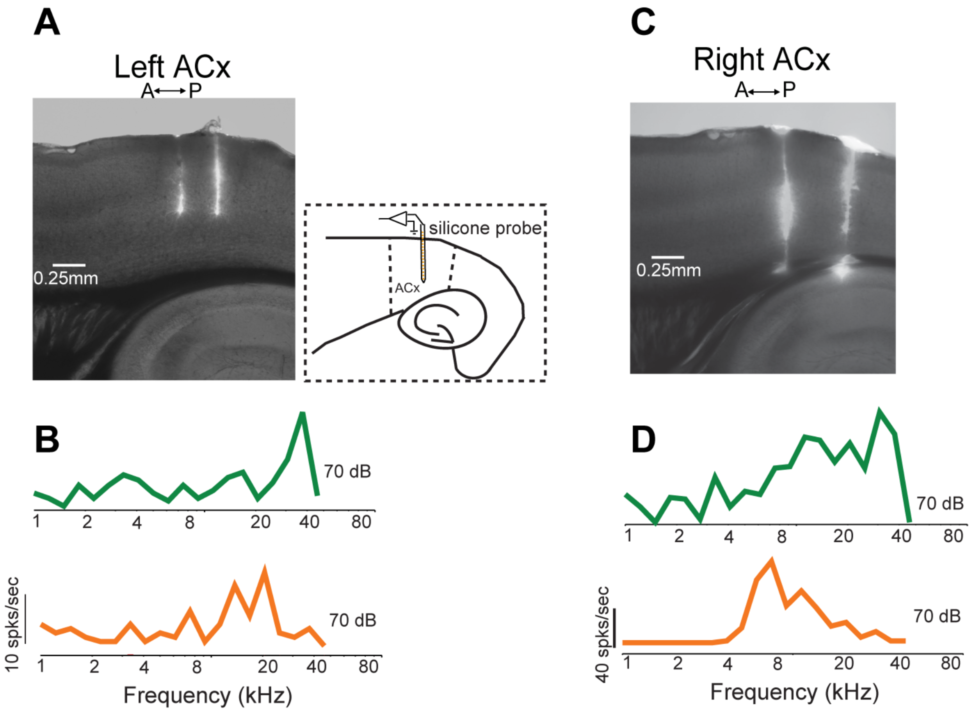

Supplementary Figure 3.

Relationship between cortical distance and tonotopy in the ACx. Translaminar multiunit silicone probe recordings showing the DiI tracks of the penetrations (top) and peristimulus time histograms of tone-evoked responses (bottom). We found that one-octave change in frequency preference occurs over the span of 0.275 mm of cortical space (**A-B**), and approximately a three-octave change occurs in 0.5 mm (**C-D**).

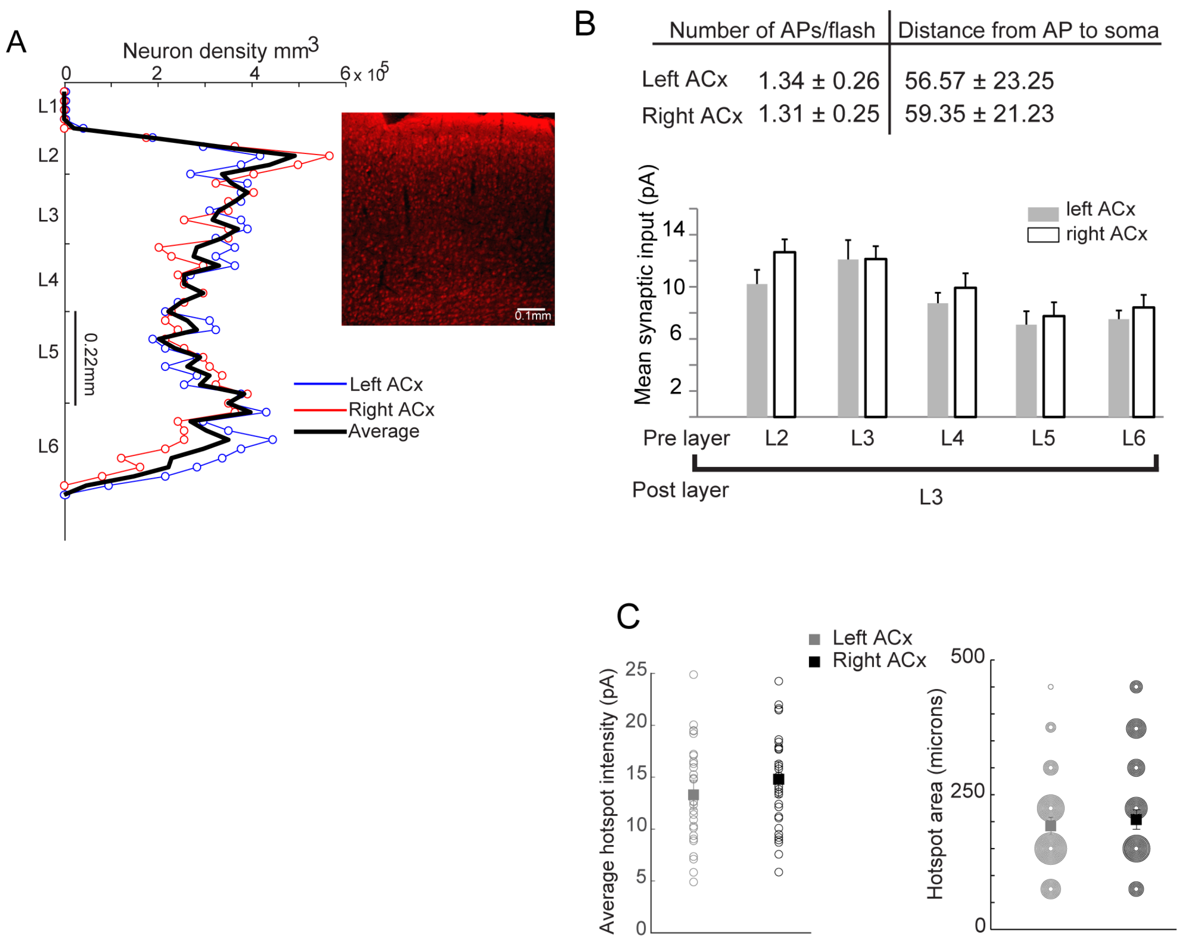

Supplementary Figure 4

Summary of anatomical and functional properties that do not differ between the left and right ACx. (**A**) Average neuronal density showing there was no significant difference in density between the left and right ACx. Slices were stained with the neuronal marker Neun (inset image, n=2 mice) and confocal stacks were analyzed with the ImageJ particle analysis plug in. (**B, top**) Neuronal excitability does not differ between the left and right ACx. We performed excitation profiles of neurons in all layers of the left and right ACx (n=20 for each hemisphere). The table shows that the number of action potentials (APs) evoked per UV flash and distance from soma an AP was evoked did not differ between the left and right ACx. (**B, bottom**) The average presynaptic input to Layer 3 did not differ between the left and right ACx. (**C, left**) The average hotspot intensity was not significantly different between the left ($n = 38$) and right ($n = 40$) ACx ($p = 0.2706$, Wilcoxon rank-sum.) (**C, right**) The average hotspot area was not significantly different between the left ($n = 38$) and right ($n = 40$) ACx ($p = 0.379$, Wilcoxon rank-sum.) The number of circles represents the number of cells with that hotspot area. All data are presented as mean±SEM.

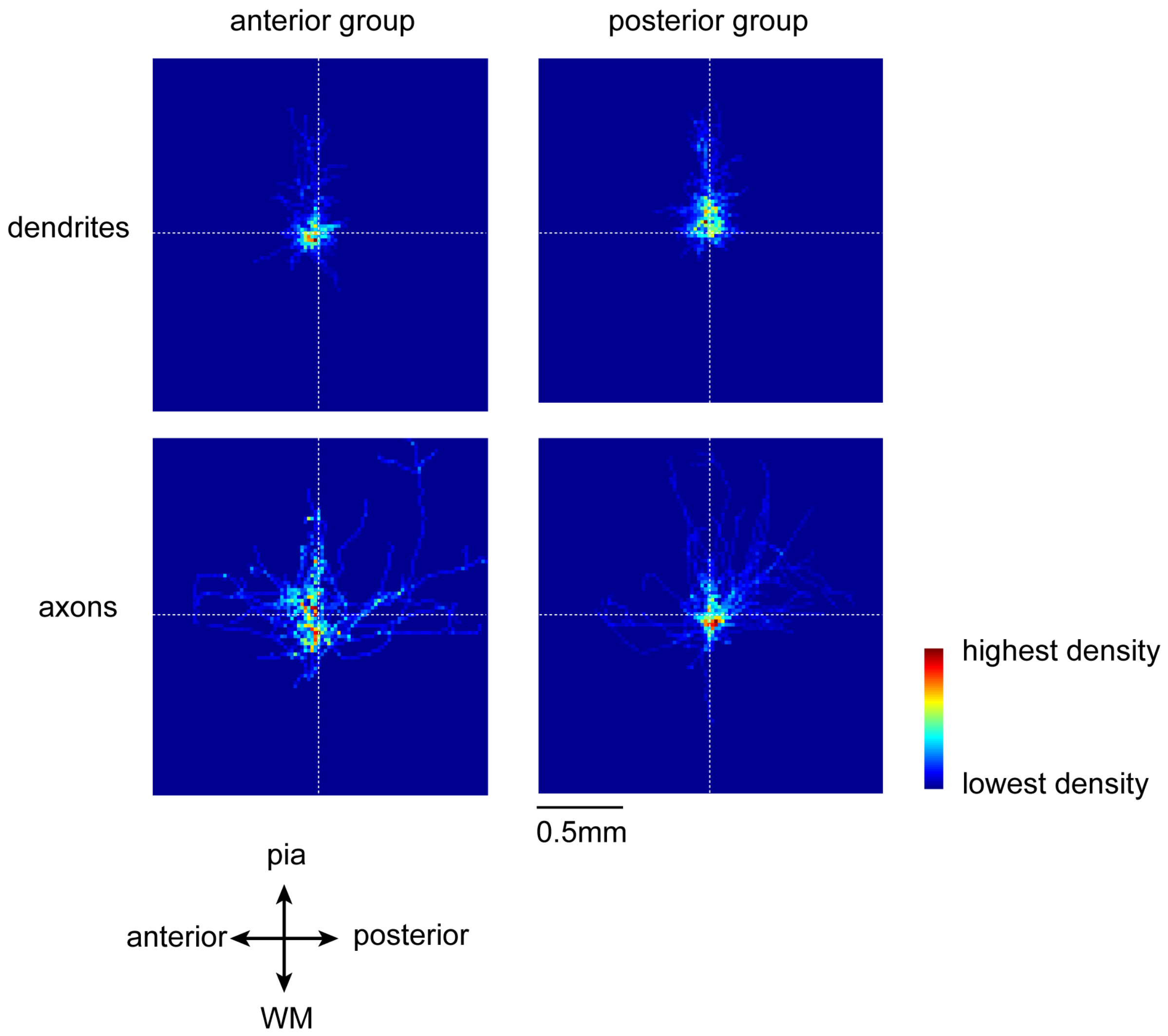

Supplementary Figure 5

Summed cartesian maps of process length density for reconstructed anterior (n=4) and posterior (n = 5) cells. Maps were made from reconstructed, biocytin-filled cells from in vitro slices as described in the main Methods. Process length was calculated as microns of axon/dendrite per cubic micron of cortex (c.f. Levy and Reyes 2012, Stepanyants and Chklovskii, 2005), then projected onto the 2D plane indicated by the labeled axes. The process density scale (color bar) was normalized to the highest value for each of the four maps, for ease of comparing the overall patterns. Crosshairs indicate the position of the soma, which was aligned for all cells in each group. The polar histograms in figure 5B, C of the main text are derived from the same raw data.

	high dendrites	high axons	low dendrites	low axons	normal dendrites	normal axons	total		fraction cut: dendrites	axons
**posterior group**										
									0.16666667	0.27272727
Cell 1	5	13	0	2	25	40	85		0.42647059	0.31578947
Cell 2	23	10	6	2	39	26	106		0.15	0.10204082
Cell 3	6	4	0	1	34	44	89		0.0862069	0.14545455
Cell 4	5	7	0	1	53	47	113		0.09803922	0.05084746
Cell 5	5	3	0	0	46	56	110			
								**mean**	**0.18547667**	**0.17737191**
								**standard dev.**	**0.13891569**	**0.11286496**
**anterior group**										
									0.23076923	0.29347826
Cell 1	11	18	1	9	40	65	144		0.2	0.06930693
Cell 2	12	6	0	1	48	94	161		0.18604651	0.17777778
Cell 3	8	4	0	4	35	37	88		0.11627907	0.28947368
Cell 4	5	12	0	10	38	54	119			
								**mean**	**0.1832737**	**0.20750916**
								**standard dev.**	**0.04841337**	**0.10660313**

Supplementary Table 4

Effect of slicing on cell intactness. Biocytin-filled cells from *in vitro* 300 μm slices were reconstructed with Neurolucida (see main Methods) and axonal and dendritic branch endings were classified as either intact (ending within the thickness of the slice) or cut high (at the upper cut surface of the slice, as it was oriented in the recording chamber) or low (at the lower cut surface). The preponderance of high cuts reflects the fact that cell bodies were generally 50-100 μm from the upper surface (62 +/- 12 μm, n = 9). The cut fraction (right-most columns) was calculated as (high+low / high+low+intact) Differences in mean values between the posterior and anterior groups were not statistically significant (p = 0.7, unpaired two-tailed t-test).

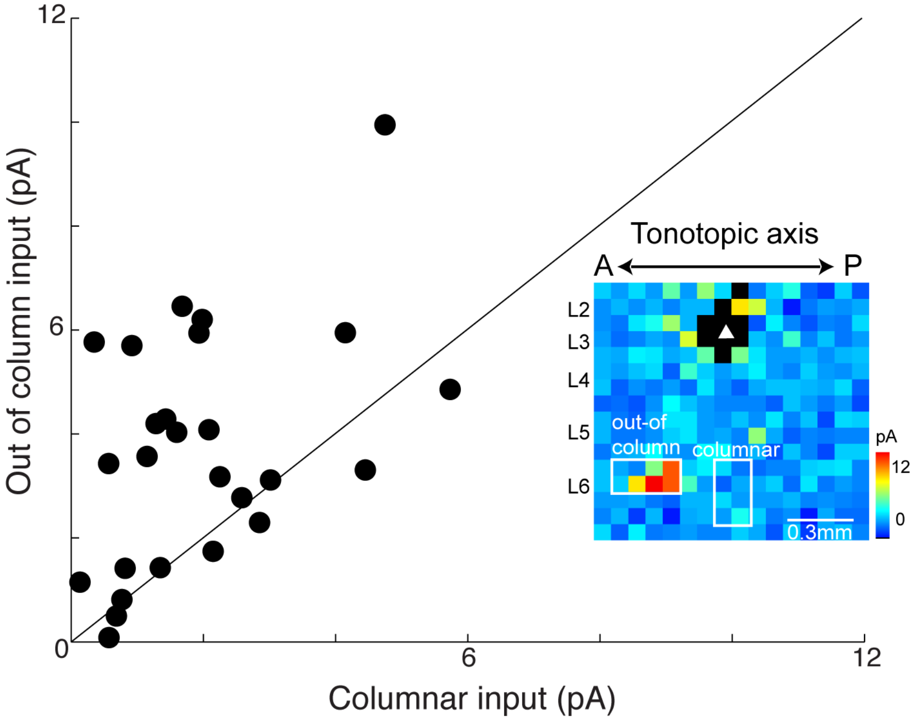

Supplementary Figure 6

Comparing the strength of columnar and out-of-column input using Laser Scanning Photostimulation. All points are paired data from the same cell. The cells shown are from the middle and posterior clusters of the left ACx (Figure 4C). Inset depicts the mapped regions used to measure these inputs. Most of the data points are above the line of unity, indicating that the out-of-column pathway is significantly stronger than the columnar ($p = 0.0036$, paired t-test, $n = 26$ cells, 18 mice).

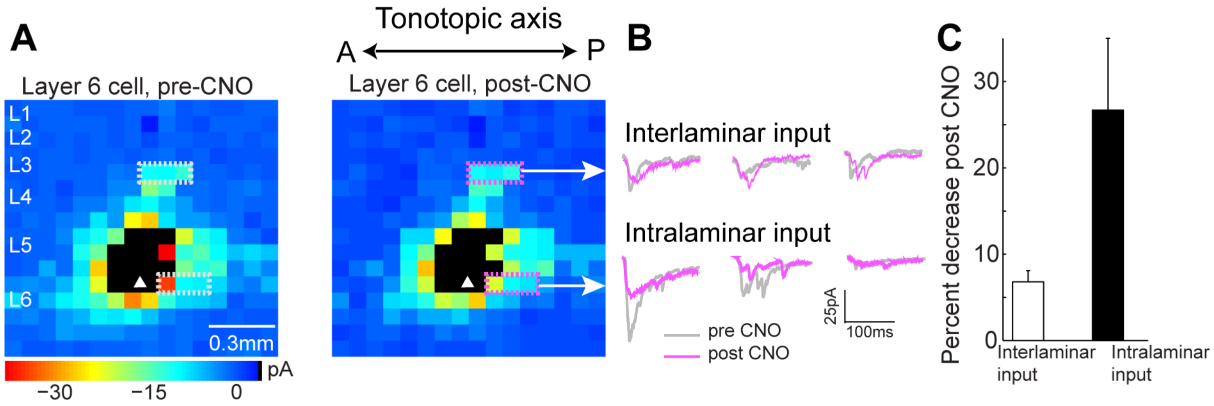

Supplementary Figure 7

Impact of silencing L6-Ntsr1 input on synaptic input maps using Laser Scanning Photostimulation and chemogenetics. (**A**) Single cell map from one L6-Ntsr1 neuron before (left) and after (right) CNO infusion into the bath. (**B**) Adding CNO to the bath largely reduced the amplitude of intralaminar L6 input compared to interlaminar input (boxed regions in A). (**C**) There was a significant difference in the percent decrease between inter and intralaminar inputs ($p = 0.038$, $n = 5$ cells, 5 mice Wilcoxon rank-sum.) All data are presented as mean ± SEM.
